# The metaverse in orthopaedics: Virtual, augmented and mixed reality for advancing surgical training, arthroscopy, arthroplasty and rehabilitation

**DOI:** 10.1002/ksa.12723

**Published:** 2025-07-07

**Authors:** Mahmut Enes Kayaalp, Efstathios Konstantinou, Bedri Karaismailoglu, Gian Andrea Lucidi, Mehmet Kaymakoglu, Romed Vieider, Joseph D. Giusto, Jumpei Inoue, Michael T. Hirschmann

**Affiliations:** ^1^ Department of Orthopaedics and Traumatology Istanbul Fatih Sultan Mehmet Training and Research Hospital University of Health Sciences Istanbul Türkiye; ^2^ Faculty of Health Sciences Brandenburg, Brandenburg Medical School Theodor Fontane Germany; ^3^ Department of Orthopaedic Surgery and Musculoskeletal Trauma University Hospital of Larisa, School of Health Sciences University of Thessaly Larisa Greece; ^4^ CAST (Cerrahpasa Research, Simulation and Design Laboratory) Istanbul University‐Cerrahpasa Istanbul Türkiye; ^5^ Department of Orthopaedics and Traumatology Cerrahpasa Faculty of Medicine Istanbul University‐Cerrahpasa Istanbul Türkiye; ^6^ II Clinica, IRCCS Istituto Ortopedico Rizzoli Bologna Italy; ^7^ Department of Orthopedics and Traumatology Faculty of Medicine Izmir University of Economics Izmir Türkiye; ^8^ Department of Orthopaedic Surgery University of Pittsburgh Medical Center Pittsburgh Pennsylvania USA; ^9^ Department of Sports Orthopaedics Technical University of Munich Munich Germany; ^10^ Department of Orthopaedic Surgery Nagoya Tokushukai General Hospital Kasugai Aichi Japan; ^11^ Department of Orthopedic Surgery and Traumatology Kantonsspital Baselland Bruderholz Switzerland; ^12^ Department of Clinical Research Research Group Michael T. Hirschmann, Regenerative Medicine & Biomechanics University of Basel Basel Switzerland

**Keywords:** augmented reality, education, medical, graduate, orthopedic procedures, rehabilitation, virtual reality

## Abstract

**Purpose:**

The metaverse and extended reality (XR), which includes augmented reality (AR), virtual reality (VR) and mixed reality (MR), are transforming orthopaedic surgery by enhancing training, procedural accuracy and rehabilitation. However, a literature review of these new virtual tools is lacking. The purpose of this narrative review is to summarise available evidence about the metaverse and discuss current and future clinical applications.

**Methods:**

A narrative review of the current literature was performed for studies evaluating XR tools and their respective clinical and educational utility. Studies from all orthopaedic subspecialties were eligible for inclusion. The XR tools evaluated in each study were categorised according to the reality spectrum and future research or clinical applications were discussed.

**Results:**

XR is a technological spectrum that includes AR, VR and MR to create immersive and interactive surgical training environments. VR‐based simulators may improve surgical education by allowing trainees to refine their skills in a risk‐free setting. AR may enhance intraoperative guidance and has been studied within orthopaedics to improve implant positioning accuracy and reduce complications in procedures including arthroscopy and total joint arthroplasty. In rehabilitation, AR and VR have been implemented to facilitate patient engagement and adherence, promoting functional recovery through gamified therapy and remote telerehabilitation.

**Conclusions:**

There has been a paradigm shift in orthopaedic care in which digital tools are integrated with patient care to optimise patient outcomes. However, challenges to the widespread implementation of promising XR technology include high costs, steep learning curves and limited clinical validation. Ethical concerns, including data security and patient privacy, further complicate its use in clinical settings. Future research must focus on cost‐effectiveness, standardisation and improving accessibility to ensure seamless integration into clinical practice.

**Level of Evidence:**

Level V.

**Abbreviations**
3D: three‐dimensional
AR: augmented reality
HMD: head‐mounted displays
MIS: minimally invasive surgery
MR: mixed reality
TKA: total knee arthroplasty
VR: virtual reality
XR: extended reality

## INTRODUCTION

**Figure 1:**
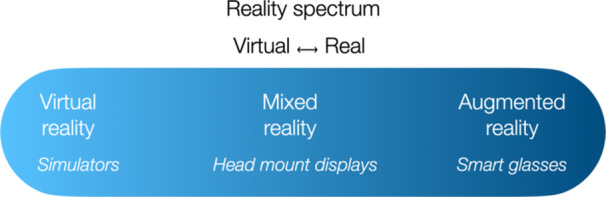
illustrates the reality spectrum from a fully virtual to real environment. Mixed reality bridges virtual and augmented reality using devices such as head‐mounted displays, whereas simulators are examples of virtual reality (VR) and smart glasses examples of augmented reality (AR).

The term 'metaverse', first conceptualised in 1992, represents an immersive three‐dimensional (3D) virtual ecosystem that merges augmented physical reality with persistent synthetic environments [32]. At its core, the metaverse integrates extended reality (XR) technologies, which include virtual reality (VR), augmented reality (AR) and mixed reality (MR), to facilitate dynamic interactions across virtual and physical spaces (Figure 1). VR can be classified on the increasingly virtual end of the spectrum as these technologies immerse users in entirely digital environments. Conversely, AR lies on the opposite end of the spectrum in which technologies overlay contextual digital data onto real‐world settings to enhance spatial perception. MR bridges these opposite ends of the XR spectrum, enabling simultaneous interaction with physical and virtual elements [18, 27]. Together, XR modalities transcend traditional digital interfaces, enabling collaborative, multisensory engagement (Figure 1).

In healthcare, the metaverse and XR technologies are driving a paradigm shift in which the merging of virtual and physical domains has changed medical training, procedural accuracy and direct patient care. For example, VR simulators have previously demonstrated efficacy in surgical skill acquisition while also minimising the risks associated with early clinical exposure and avoiding the use of cadavers [1, 35]. Within orthopaedic surgery specifically, AR navigation systems enhance procedural accuracy in total knee and hip arthroplasty by improving component alignment and reducing intraoperative errors, which may lead to improved long‐term patient outcomes [46]. These advancements signal a paradigm shift: the metaverse is not merely a tool but a holistic framework for bridging gaps between orthopaedic training, surgical execution and patient outcomes. Among these innovations, digital twin systems represent a cutting‐edge application of XR in orthopaedics, enabling personalised surgical planning, real‐time rehabilitation monitoring and predictive modelling of musculoskeletal conditions through the integration of multimodal data and computational modelling [11]. In parallel with XR advancements, large language models such as ChatGPT and emerging multimodal artificial intelligence (AI) systems like DeepSeek are also influencing orthopaedic scholarship—enhancing language accessibility, streamlining manuscript preparation and offering novel tools for clinical decision support and literature navigation [9, 23, 24]. The integration of AI systems in XR tools would further improve orthopaedic surgery through personalised and predictive modelling and assist in surgeon training [31].

Within the subspecialty of sports traumatology and arthroscopic surgery, XR technologies provide demonstrable and clinically relevant benefits. VR‐based arthroscopy simulators allow trainees to repeatedly practice shoulder and knee procedures in risk‐free environments—accelerating the learning curve while improving safety. Intraoperatively, AR‐assisted navigation can enhance tunnel positioning in ACL reconstruction, reduce fluoroscopy time during multiligament repairs and improve visualisation in complex shoulder procedures. These innovations have the potential to reduce technical errors, enhance the accuracy of graft positioning and contribute to better long‐term outcomes in athletes. In the postoperative setting, virtual and AR–based rehabilitation tools can support greater patient adherence, facilitate remote supervision and assist in more efficient return‐to‐sport decision‐making following ligament or cartilage injuries. As these technologies continue to evolve and demonstrate clinical value, their integration into both operative workflows and sports rehabilitation protocols is expected to expand.

While numerous publications have described XR technologies individually, this review distinguishes itself by offering a structured, orthopaedic‐specific synthesis. It consolidates current knowledge and provides a clinician‐oriented perspective on how XR integration can enhance procedural outcomes, patient safety and accessibility in orthopaedic care. Focusing on three pillars—(1) immersive surgical education and VR simulation, (2) AR/MR‐enhanced surgical procedures and (3) rehabilitation—we critically evaluate clinical evidence and emerging trends to demonstrate how the convergence of virtual and physical realities is redefining standards of care and highlight the challenges that must be addressed for sustainable implementation. It is hypothesised that recent advances in XR technology have been adopted in orthopaedics, enhancing surgical education, planning, procedures and postoperative rehabilitation.

### Orthopaedic surgical training and the role of immersive technologies

**Figure 2:**
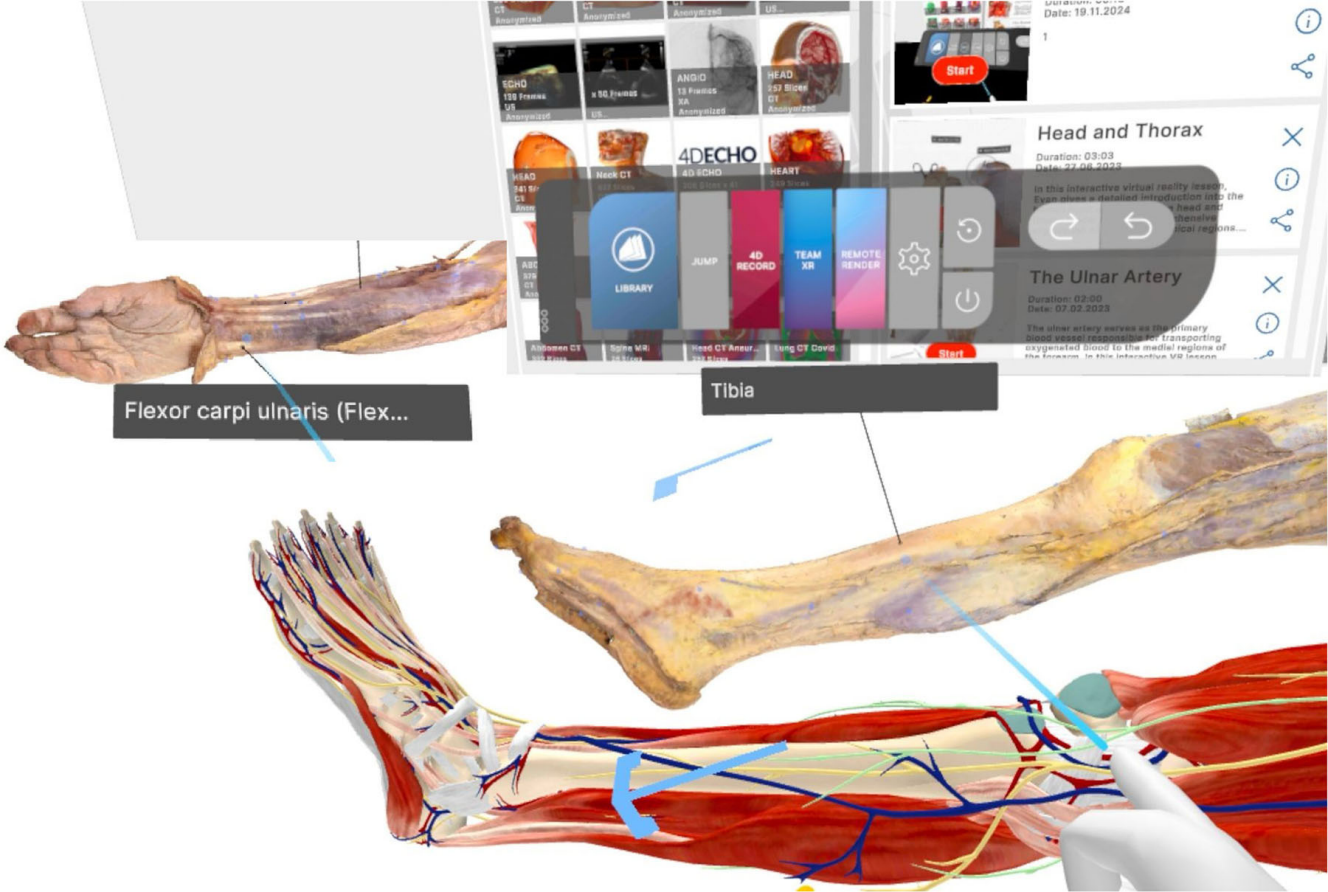
Example of anatomy education using three‐dimensional (3D) anatomical models and 3D‐scanned cadaveric dissections. Dissection Master XR (Medicalholodeck, Zurich, Switzerland).

The concept of utilising VR‐based simulators for surgical training originated from the observation that surgeons who had experience playing video games adapted more quickly to tasks requiring indirect visualisation and fine motor coordination on a two‐dimensional screen—particularly in arthroscopic and laparoscopic surgeries [8, 15, 17, 21]. VR‐based simulators have since emerged as transformative tools in surgical training. They allow trainees to practice a wide range of skills—from anatomical dissections (Figure 2) to complex surgical procedures such as arthroscopic techniques, fracture fixation and arthroplasty—in risk‐free, controlled environments. This immersive practice may ultimately enhance technical precision and help reduce intraoperative errors in the future [4, 41]. Table 1 summarises key distinctions between traditional orthopaedic training and XR‐enhanced approaches, based on the authors' synthesis of current literature and clinical perspectives.

**Table 1:** Main differences between traditional and immersive technology enriched orthopaedic surgical training.

Aspect of education	Traditional orthopaedic training	Immersive technology enriched training
Educational methods	Mainly passive (lectures, webinars, observation‐based training in OR	Highly interactive (hands‐on learning, repeated procedures
Patient safety	Trainee‐patient interaction since early training	Risk‐free simulators without patient involvement
Learning feedback	Delayed, Mostly instructor dependent	Immediate, Instantaneous error correction
Learning engagement	Generally moderate	High engagement
Learning curve	Variable (clinical availability, instruction experience)	Reduced (repetitive and standardised scenario)
Accessibility and repeatability	Restricted (clinical volume, case variability)	Widely accessible (standard virtual scenarios)

Abbreviation: OR, operating room.

VR simulators can be classified into two based on the use of VR headsets;

- Screen‐based VR simulators
  - These simulators combine physical models, haptic feedback and high‐resolution screens to simulate real‐life procedures. They often use tracked instruments but do not rely on VR headsets.

- Fully immersive headset‐based VR simulators
  - These systems use VR headsets and hand controllers to create an immersive training experience that rely heavily on virtual environments rather than physical models.

Screen‐based VR simulators improve hand‐eye coordination and procedural accuracy in knee and shoulder arthroscopy, allowing surgeons to improve their technique before performing actual surgeries [33, 39]. Research indicates that these simulators may offer improved surgical competence with trainees who used VR demonstrating superior proficiency in complicated operations compared to those undergoing conventional training [5]. A randomised controlled trial found that VR simulation significantly improved arthroscopic skills compared to bench‐top simulation practice [3]. A systematic review further supports the efficacy of VR in improving psychomotor skills and surgical performance across various orthopaedic specialties, including trauma, arthroplasty, arthroscopy and spine surgery [41]. Additionally, some VR‐based training platforms incorporate haptic feedback, which provides tactile resistance and sensation during surgical simulations. This technology helps trainees develop a more nuanced understanding of soft tissue handling, force application and instrument manipulation. A study suggested that haptic‐enabled VR systems can reduce operation time and tissue damage while improving surgical accuracy [6].

**Figure 3:**
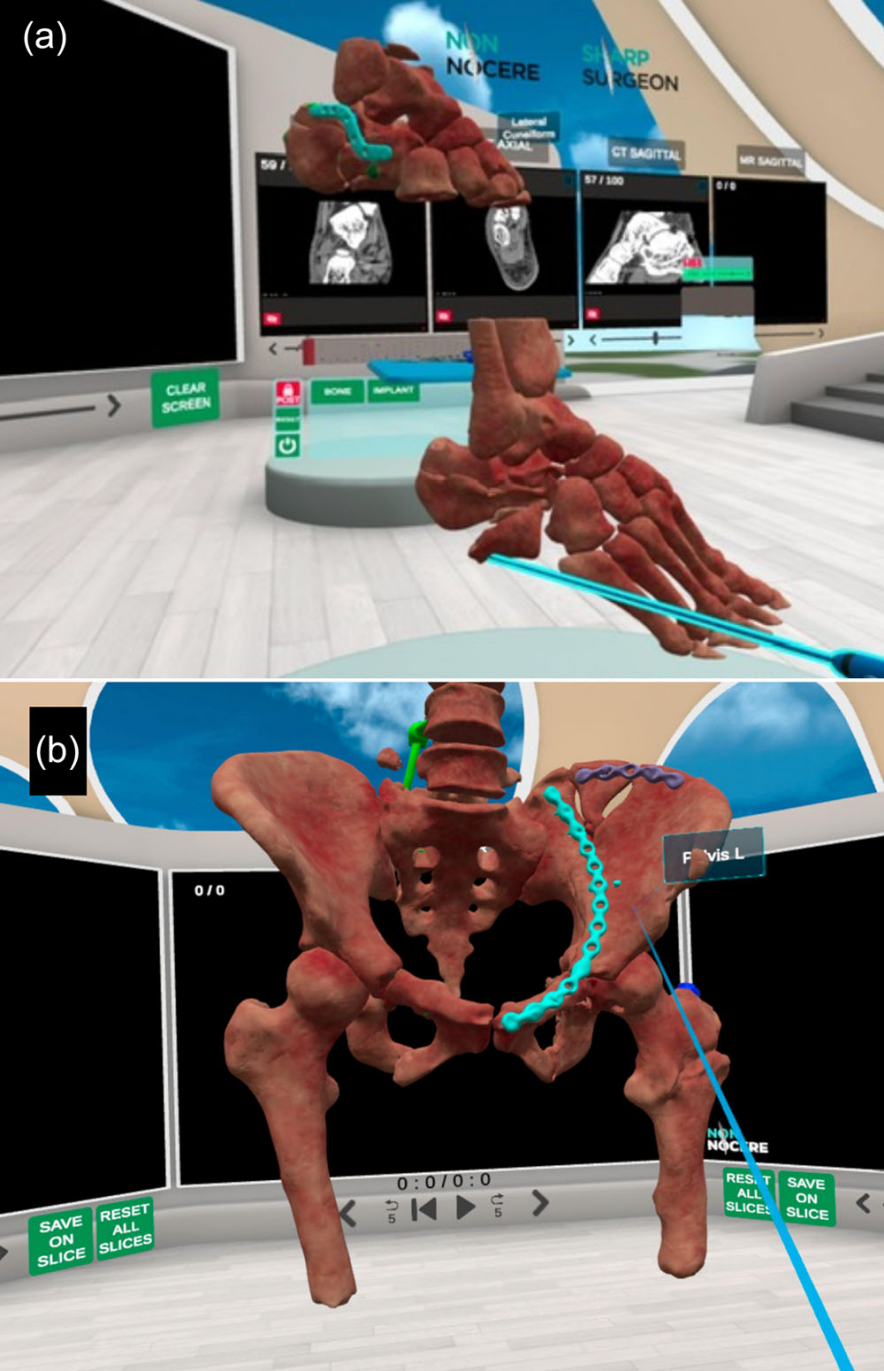
Example virtual reality (VR) education modules for orthopaedic trauma surgery. (a). Fracture reduction tool in a calcaneal fracture model. (b). Plate‐screw application in a pelvis fracture model. SharpSurgeon (Non Nocere GmbH, Berlin, Germany).

**Figure 4:**
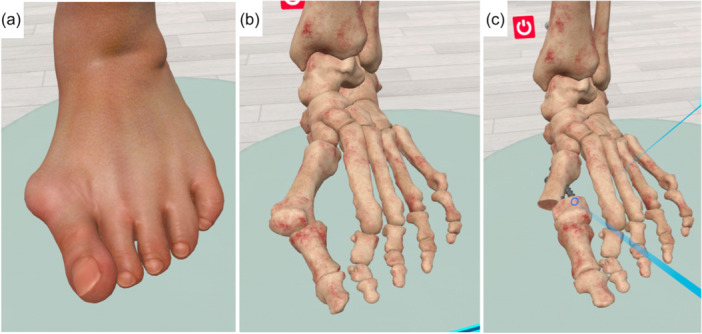
Minimal invasive hallux valgus surgery education module. (a) Preoperative deformity evaluation on a complete foot model. (b) Deformity evaluation on bone model of the same patient. (c) Postoperative view of corrected deformity after osteotomy and screw fixation. SharpSurgeon (Non Nocere GmbH, Berlin, Germany).

**Figure 5:**
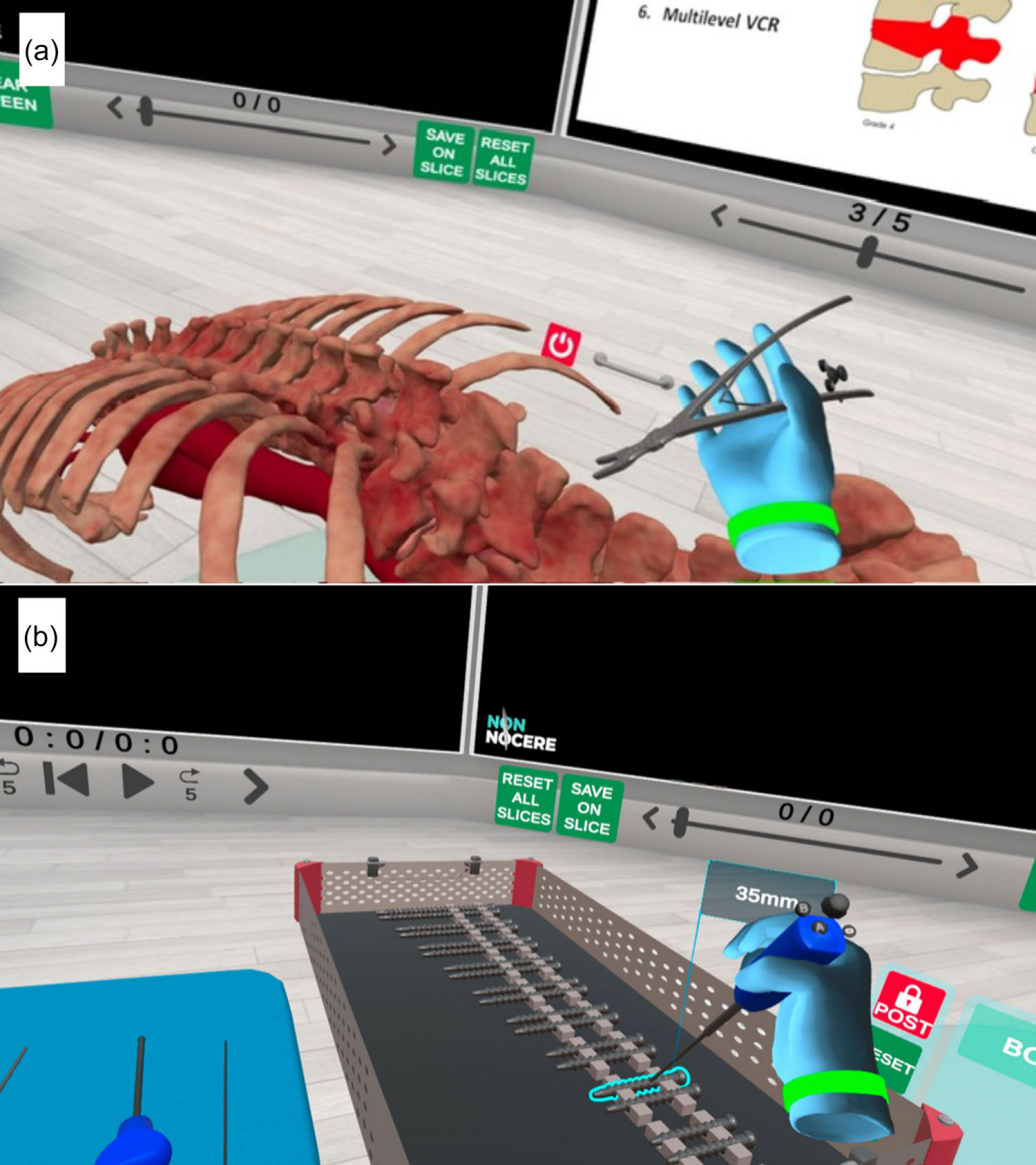
Example of using virtual reality to practice use of surgical instruments and implants. (a) Rongeur application in spine surgery. (b) Screw application using a screw‐driver. SharpSurgeon (Non Nocere GmbH, Berlin, Germany).

**Figure 6:**
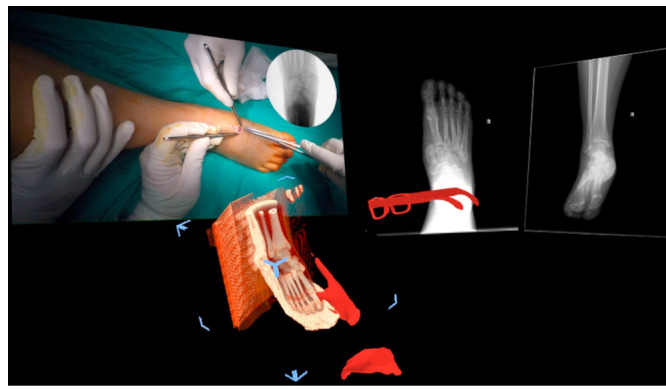
Real‐time virtual reality collaborative session on the surgical treatment of Lisfranc injuries, enabling educators and trainees from around the world to participate. Medical Imaging XR (Medicalholodeck, Zurich, Switzerland).

While most screen‐based VR simulators are designed for arthroscopic procedures, other applications—such as fracture fixation (Figure 3a,b), deformity correction and arthroplasty simulations—are increasingly available, particularly in fully immersive headset‐based systems [4, 33, 35]. These immersive platforms can provide step‐by‐step surgical instructions, enable the completion of entire procedures (Figure 4a–c), and allow the use of virtual instruments and implants (Figure 5a,b). They also support real‐time collaborative sessions, allowing participants from around the world to join, thereby facilitating global surgical education (Figure 6). A recent randomised controlled trial found that VR training for unicompartmental knee arthroplasty resulted in surgical competence equivalent to that achieved with traditional technique guides, suggesting that VR modalities may be valuable educational adjuncts [50]. VR‐based telecommunication platforms can also enhance surgical education by facilitating real‐time collaboration between trainees and specialists across different geographic locations [18].

AR and MR require specialised devices to integrate virtual components into real‐world settings, including a position tracker and a display. Devices designed for this purpose include glasses and headsets or head‐mounted displays (HMDs). These devices allow for intraoperative image guidance, data monitoring and surgical training [2, 35, 36], although limitations such as low pass‐through resolution have been reported [2].

Currently, VR and AR technologies are incorporated into surgical training programs, offering interactive educational experiences. VR simulations provide an enhanced view of anatomical structures, improving spatial awareness and technical precision during procedural training [18]. Simulator platforms also offer real‐time feedback, which provides trainees the opportunity to refine their skills before participating in live surgeries [1]. Additionally, AR technology has been shown to be effective in surgical training, providing an experience comparable to wet laboratory tutorials but with greater trainee engagement [36]. Additional advantages of VR‐training include the opportunity to consistently practice various standardised clinical scenarios without waiting for specific clinica cases, as well as eliminating variability associated with mentor availability, expertise and potential bias toward a preferred surgical approach [45].

HMDs also enable hands‐free access to surgical guidance and remote mentorship, expanding the ability for surgeons to provide expert instruction during procedures [33]. A recent systematic review emphasised the role of XR technologies in reshaping orthopaedic education by improving technical performance, reducing learning curves and facilitating remote training opportunities [31]. The integration of VR and AR into surgical curricula continues to evolve, with ongoing research exploring new applications to optimise training and enhance patient safety. However, concerns remain regarding the real‐world benefits of VR devices, particularly in justifying their cost and the associated institutional burdens [4].

The metaverse offers several potential benefits, including increased student engagement, safe environments for decision‐making and the ability to incorporate gamification. Additionally, blockchain technology can be utilised to verify and authenticate training activities. In the future, increasingly refined and standardised virtual training could enhance the learning experience by evolving from a single‐user interaction to a highly realistic and complex simulations. Such structured training could potentially become an integral part of the core curriculum in orthopaedic surgical education [10]. However, challenges such as disparities in digital access, the high cost of AR/VR devices and platform fragmentation may hinder widespread adoption [42].

### Applications in orthopaedic surgery

The integration of VR and AR into orthopaedic surgery has revolutionised preoperative planning, intraoperative navigation and postoperative rehabilitation. By providing interactive, immersive visualisation tools, these technologies have been shown to improve surgical precision, reduce operative time and enhance patient outcomes [31]. Table 2 summarises the current applications and benefits of VR and AR in orthopaedic surgery. Where applicable, representative commercial platforms have been included to enhance the table's practical relevance. These examples illustrate the types of XR solutions currently available and their alignment with specific clinical needs, though they are not intended to be exhaustive.

**Table 2:** Current applications of virtual reality (VR) and augmented reality (AR) in orthopaedic surgery.

Surgical phase or procedure technology	Technology	Orthopaedic application	Potential benefits	Example platforms/providers
Preoperative planning	VR	Fracture fixation, deformity correction	Improved precision, reduced time, minimise radiation	SharpSurgeon, ImmersiveTouch
Intraoperative navigation	AR	Screw, plate, intramedullary nail distal locking and guidewire placement	Increased accuracy, reduced time, reduced radiation exposure	Microsoft HoloLens 2, Proximie
Arthroscopy	AR	Shoulder and Knee Arthroscopy	Real‐time anatomical visualisation, minimise errors	Osso VR, Surgical Theater
Total joint arthroplasty	AR	Total Knee and Total Hip Arthroplasty	Precise implant positioning, improved alignment	HoloLens 2, ImmersiveTouch

Abbreviations: AR, augmented reality; VR, virtual reality.

The organisation of Table 2 is designed to align with the key stages of orthopaedic surgical care where XR technologies have demonstrated distinct clinical value. These stages include preoperative planning, intraoperative guidance and specific procedural applications such as arthroscopy and arthroplasty. This structure enables a clear understanding of how different XR modalities contribute to different phases of care delivery.

VR systems are widely utilised in orthopaedic trauma procedures, especially for fracture fixation and preoperative planning. Studies suggest that VR‐based simulations enhance intraoperative fracture reduction efficiency, thereby improving overall surgical performance and reducing radiation exposure by decreasing reliance on fluoroscopic guidance [47].

In contrast, AR is more commonly employed intraoperatively, especially for surgical navigation [19, 26]. AR‐assisted techniques have demonstrated improved accuracy in screw and plate placement, potentially enhancing mechanical stability and accelerating recovery [31]. These technologies also aid in guidewire placement for fracture management [19, 48], distal locking of intramedullary nails [12], and pelvic screw placement [40] by incorporating a virtual image projection into the surgeon's visual field and utilising magnetic guidance (Supporting Information: Figure 1). Such applications offer an immersive intraoperative experience and may shorten surgical time and radiation exposure [19].

AR‐based overlays can also enhance real‐time visualisation of anatomical structures during shoulder arthroscopy, minimising procedural errors and improving efficiency [31]. In total knee arthroplasty (TKA), AR‐assisted navigation improves component alignment accuracy, reducing the risk of malpositioning and optimising implant longevity [33]. Similarly, AR‐guided hip arthroplasty allows for real‐time visualisation of acetabular cup positioning, leading to more precise implant placement and lower complication rates [31]. In shoulder arthroplasty, VR‐based preoperative simulations provide surgeons with enhanced spatial awareness, which translates to improved surgical outcomes [47].

When combined with AI, VR/AR technologies—collectively known as XR—have the potential to provide rapid, adaptive solutions to emerging challenges in surgical planning and intraoperative decision‐making. AI can enhance XR applications through personalised and predictive modelling and assist in surgeon training. While AI is already well established in diagnostics, its integration with XR in orthopaedic surgery is still in early stages, with several trials underway [31]. This combination may also help bridge the experience gap between novice and expert surgeons. However, significant challenges remain, including ethical concerns related to the use of large patient datasets and the risk of limited performance in rare or data‐scarce situations [43].

The ongoing incorporation of VR and AR in orthopaedic surgery is expected to improve surgical precision, reduce complications and improve patient safety. Additionally, AR improves visualisation in minimally invasive surgery (MIS) by overlaying critical anatomical structures, minimising the need for excessive soft tissue dissection. However, ongoing research is essential to optimise these technologies for routine clinical application, particularly given their high costs and the lack of definitive evidence supporting their cost‐effectiveness [47].

### Use in musculoskeletal rehabilitation

XR‐based rehabilitation extends musculoskeletal care beyond the confines of traditional clinical settings through the integration of telemedicine platforms and wearable technologies. Among these, inertial measurement units (IMUs) have emerged as a pivotal component of AR/VR‐based recovery programs, offering objective, continuous monitoring of joint kinematics and gait parameters. When affixed to the limb, IMUs capture motion data with a high degree of fidelity, enabling real‐time transmission to digital platforms or healthcare providers. Recent studies have demonstrated that modern IMU systems yield knee motion metrics comparable in accuracy to those obtained in laboratory‐based motion capture environments, while allowing for assessment in ecologically valid, real‐world contexts [22]. These wearable technologies facilitate personalised, remote rehabilitation protocols, particularly following procedures such as total knee arthroplasty. Technological advances over the past decade have revolutionised musculoskeletal rehabilitation. The use of telemedicine for postoperative follow‐ups and rehabilitation has optimised resource utilisation without compromising patient recovery [7, 14, 25]. The integration of VR and AR in orthopaedic rehabilitation further enhances patient compliance and functional recovery by offering interactive and engaging rehabilitation sessions. VR creates a fully immersive digital environment that replaces the real world, requiring patients to wear HMDs. In contrast, AR overlays digital elements onto the real‐world environment, allowing patients to see both their physical surroundings and virtual guidance through smart glasses, tablets, or HMDs. This capability enables real‐time feedback and guidance during exercises. A systematic review highlighted the potential for AR‐based rehabilitation to improve post‐injury functional outcomes through guided, interactive movement therapy [49].

VR‐based rehabilitation programs have proven particularly beneficial for patients recovering from musculoskeletal injuries and joint replacement surgeries. Studies indicate that gamified rehabilitation environments reduce pain perception, enhance range of motion and improve functional recovery by promoting active patient participation [28]. In anterior cruciate ligament reconstruction, AR‐assisted telerehabilitation has been shown to be as effective as in‐person physiotherapy, with comparable functional improvements in patients and higher compliance rates [29].

VR‐assisted rehabilitation has also been applied for chronic musculoskeletal disorders including low back pain, which has been shown to significantly reduce pain‐related fear and disability scores [30]. The ability to conduct therapy remotely through VR and AR platforms also ensures continuity of care, particularly for patients facing logistical barriers to in‐person rehabilitation [13].

VR‐based applications have demonstrated clinical benefits in other rehabilitation programs as well, including reducing acute procedural pain during hand therapy [20]. Additionally, XR devices incorporating HMDs have been proposed for managing phantom limb pain in patients with acquired amputations, which may offer therapeutic benefits [16]. As the application of XR technologies in rehabilitation continues to expand, future research should focus on optimising treatment protocols, tailoring interventions to individual needs and assessing long‐term outcomes to establish their effectiveness in routine orthopaedic practice [31].

## DISCUSSION

XR technologies, encompassing VR, AR and MR, are increasingly influencing orthopaedic surgery by enhancing surgical precision, education and patient recovery. This review has outlined their current applications in immersive training, intraoperative guidance and musculoskeletal rehabilitation. However, the clinical integration of XR tools presents certain challenges that warrant careful consideration.

### Anatomical considerations and technical challenges

While XR platforms provide enhanced visualisation and navigation, anatomical complexity introduces notable limitations [37]. Joints such as the shoulder, hip and foot exhibit intricate geometries and variable morphology, demanding precise, patient‐specific modelling. Intraoperative AR overlays may be affected by soft tissue distortion or registration drift, particularly during minimally invasive procedures. In arthroscopy, limited field of view and reliance on indirect visualisation may reduce the effectiveness of XR‐enhanced guidance. Moreover, current head‐mounted display systems often face ergonomic limitations and visual resolution constraints, affecting surgeon comfort during long procedures [2, 26, 27]. Overcoming these limitations will require ongoing improvements in device design and more sophisticated image‐processing capabilities that better adapt to surgical realities.

### Generalisability and broader clinical integration

Most of the available data on XR technologies comes from well‐funded academic centres or early adopters with access to advanced resources. As a result, it's still unclear how well these technologies will translate into everyday care, especially in rural or under‐resourced settings [44]. Even where XR devices are available, certain system limitations temper their effectiveness. For instance, most current VR simulators lack tactile (haptic) feedback and cannot recreate the full complexity of a live surgical environment [38]. Practical barriers such as cost, infrastructure gaps, digital literacy and compatibility with existing workflows all play a role in slowing adoption. In addition, many published studies are small or lack rigorous control groups, making it difficult to draw broad conclusions [44]. To truly understand how XR can impact patient care on a larger scale, future research needs to include more diverse populations and settings through well‐designed, multicenter clinical studies.

### Clinical relevance

XR technologies offer specific, high‐impact benefits across the orthopaedic care continuum. In sports traumatology and arthroscopy, VR simulators accelerate skill acquisition by offering realistic, repeatable training environments that bypass the need for cadaveric resources [1]. AR‐assisted procedures—such as ACL reconstruction, meniscal repair and labral surgery—enable improved anatomical accuracy, reduce intraoperative radiation exposure and potentially improve long‐term graft survival. Postoperatively, immersive AR/VR platforms enhance patient engagement in rehabilitation, facilitating better adherence and remote monitoring, particularly following ligamentous or chondral procedures. These benefits align with the clinical demands of orthopaedic surgery: technical precision, patient‐centred care and scalable solutions.

Recent studies further support these advantages, noting that immersive VR technologies contribute meaningfully to procedural planning, surgical training and global educational access [18]. In the context of rehabilitation, VR‐based interventions have shown significant improvements in knee function and dynamic balance following ACL injuries [7]. A recent meta‐analysis on VR rehabilitation after knee ligament reconstruction confirmed significant benefits in pain reduction and proprioception, but also noted that most underlying trials were underpowered and lacked long‐term follow‐up [34]. Incorporating such tools into routine orthopaedic practice may help translate technological innovation into tangible improvements in clinical outcomes. In summary, XR in sports traumatology and arthroscopy offers practical benefits that are directly relevant to patient care. Enhanced training means surgeons are better prepared; AR‐guided techniques mean surgeries are performed with greater accuracy; and XR‐driven rehab means athletes recover strength and confidence sooner.

## LIMITATIONS AND FUTURE DIRECTIONS

Despite the transformative potential of XR technologies in orthopaedics, significant challenges prevent its widespread adoption. High operational costs, steep learning curves for practitioners and limited clinical validation limit its use, particularly in resource‐constrained settings [33]. Ethical concerns—such as data security risks and patient privacy breaches—further complicate implementation, highlighting the need for robust systems to protect sensitive health information [13]. However, these challenges must be considered in light of the potential benefits including enhanced training programs, improved surgical precision and optimised rehabilitation outcomes [39].

The lack of standardised protocols for XR integration into clinical workflows underscores the need for rigorous, evidence‐based validation to ensure efficacy and safety [39]. Additionally, the cost of VR/AR hardware and software along with the requirement for specialised training presents further obstacles to widespread implementation [31]. The steep learning curve for healthcare professionals complicates integration, emphasising the need for structured training programs and validation studies to confirm effective utilisation [33]. Furthermore, large‐scale, multicenter trials are necessary to establish the clinical efficacy of AR/VR in rehabilitation and surgical training [29].

The future of orthopaedics will likely see advancements in AR/VR technology, leading to more precise surgical guidance, patient‐specific preoperative planning and improved rehabilitation strategies. In arthroplasty, artificial intelligence‐powered VR simulations may further enhance procedural planning and intraoperative decision‐making [49]. Telerehabilitation via AR platforms is also gaining popularity by enabling remote therapy with real‐time feedback and improving patient compliance [47]. As technology evolves, interdisciplinary collaboration between clinicians and engineers will be essential in optimising XR applications for routine clinical use. Future research should prioritise standardisation, cost‐effectiveness and ease of use to ensure seamless integration into orthopaedic practice [28].

## CONCLUSION

The metaverse and XR, with various AR, VR and MR technologies, have considerable potential to transform orthopaedic surgery by advancing residency training, surgical education, preoperative planning, intraoperative accuracy and rehabilitation. As these technologies continue to evolve, further research is crucial to confirm their effectiveness, improve cost‐efficiency, and facilitate their integration into routine clinical practice. Collaboration between clinicians and engineers will be essential to overcome current limitations and promote widespread adoption. With continued advancements, AR and VR are poised to become integral tools in modern orthopaedic surgery.

## AUTHOR CONTRIBUTIONS

All listed authors have contributed substantially to this work: All authors have read and approved the final manuscript to be submitted and published.

## CONFLICT OF INTEREST STATEMENT

M. Enes Kayaalp: Associate Editor of Knee Surgery, Sports Traumatology, Arthroscopy (KSSTA), Member of the ESSKA U‐45 Scientific Committee. Michael T. Hirschmann: consultant for Medacta, Symbios and Depuy Synthes, and is the editor‐in‐chief of Knee Surgery, Sports Traumatology, Arthroscopy (KSSTA).

## ETHICS STATEMENT

N/A.

## Supporting information


*Supplementary Figure 1. Preoperative planning using x‐ray, incorporating the virtual projection of a patient‐specific 3‐dimensional model of a Lisfranc injury into the surgeon's visual field to improve assessment of the deformity and optimize incision planning. Medical Imaging XR (Medicalholodeck, Zurich, Switzerland)*.

## Data Availability

Data available upon request (Please consult: Data Sharing Policy | Wiley).
