# Supplementary figures and images for: The metaverse in orthopaedics: Virtual, augmented and mixed reality for advancing surgical training, arthroscopy, arthroplasty and rehabilitation
Source: Knee Surg Sports Traumatol Arthrosc. 2025 Jul 7;33(8):3039–50. doi: 10.1002/ksa.12723 (PMC12310088; doi:10.1002/ksa.12723)

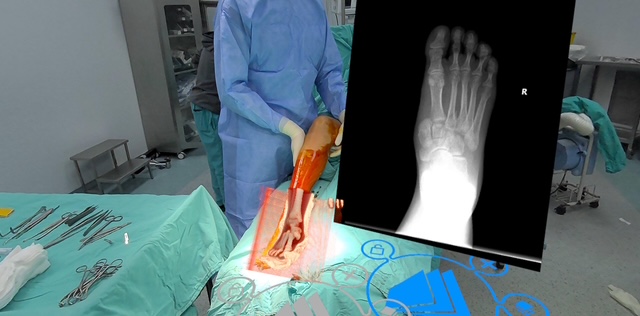

Supplement: Supplementary file 1 — Supplementary Figure 1. Preoperative planning using x‐ray, incorporating the virtual projection of a patient‐specific 3‐dimensional model of a Lisfranc injury into the surgeon's visual field to improve assessment of the deformity and optimize incision planning. Medical Imaging XR (Medicalholodeck, Zurich, Switzerland). [file KSA-33-3039-s001.jpeg]
